# Cochlear Implantation in Patients with Bilateral Sudden Sensorineural Hearing Loss after COVID-19 Infection

**DOI:** 10.3390/jpm13121708

**Published:** 2023-12-14

**Authors:** Nenad Arsović, Marija Jovanović, Snežana Babac, Ljiljana Čvorović, Nemanja Radivojević, Konstantin Arsović

**Affiliations:** 1 Faculty of Medicine, University of Belgrade, 11000 Beograd, Serbia; ljiljana.cvorovic@med.bg.ac.rs (L.Č.); nemanja.radivojevic@med.bg.ac.rs (N.R.); marija.jovanovic@med.bg.ac.rs (M.J.); 2 Institute for Otorhinolaryngology and Maxillofacial Surgery, Clinical Centre of Serbia, 11000 Beograd, Serbia; 3 Centre for Radiology and MRI, MRI Depratment Neuroradiology, Clinical Centre of Serbia, 11000 Beograd, Serbia; 4 Faculty of Special Education and Rehabilitation, University of Belgrade, 11000 Beograd, Serbia; snezanababac@fasper.bg.ac.rs; 5 ENT Clinic, Clinical and Hospital Centre ‘Zvezdara’, 11120 Beograd, Serbia

**Keywords:** COVID-19, SARS-CoV-2, profound sensorineural hearing loss, cochlear implantation

## Abstract

COVID-19 infection is associated with a variety of neurological manifestations. Since the inner ear is vulnerable to viruses, sensorineural hearing loss (SNHL) has been reported to occur following SARS-CoV-2 infection. We present here two cases of profound SNHL following SARS-CoV-2 infection. Pure-tone audiograms confirmed profound SNHL. The tympanogram and Auditory Brainstem Responses showed no abnormal symptoms. MRIs and CTs identified some changes but no significant anatomical nor physiological manifestations explaining the obvious cause for hearing loss. High doses of oral corticosteroids with additional conservative therapy were given with no therapeutic response, and therefore, cochlear implant surgery was performed. One case was bilaterally treated, and the other one received an implantation on one side. Both surgeries were carried out without intra- nor postoperative complications. Interestingly, in both cases, advanced fibrotic tissue was found during surgery. Both cases reported successful rehabilitation and are satisfied with their new sound perception following cochlear implantation.

## 1. Introduction

Almost 615 million cases of the coronavirus disease from 2019 (COVID-19) have been recorded during the pandemic. In about 6.5 million people, COVID-19 infection led to death [1]. COVID-19 infection caused by severe acute respiratory syndrome coronavirus-2 (SARS-CoV-2) is a new, under-researched disease that can have serious health consequences, regardless of the age group of affected patients.

Many different types of symptoms and signs of COVID-19 infections have been reported to date. Although the most common symptoms of COVID-19 are related to the respiratory tract, mostly cough and rhinorrhea, a number of patients reported neurological manifestations such as headaches, dizziness, neuralgia, hypogeusia, and hyposmia during infection, but these can occur several months after infection [2,3]. SARS-CoV-2 binds to the angiotensin-converting enzyme 2 (ACE2) receptor to enter human cells. Therefore, every cell expressing ACE2 may be a target to SARS-CoV-2 infection [4], like glial cells and neurons, as well as endothelial and arterial smooth muscle cells. So far, there have been several reports of sensorineural hearing loss (SNHL) in patients as one of the potential consequences of COVID-19 infections, and there is additional evidence of viral neurotropism. The mechanisms that can cause SNHL include cochlear nerve neuritis, cochleitis, immune-mediated responses, or abnormal cellular stress responses; alternatively, SNHL can be caused by ischemia, vascular occlusion, meningitis, or temporal lobe damage [5,6].

This paper describes two cases of bilateral profound SNHL with a proven presence of SARS-CoV-2 virus managed with cochlear implantation. Written informed consent was obtained from the study participants.

## 2. Materials and Methods

### 2.1. Cochlear Implant

Both subjects received the Synchrony Implant with a Standard Electrode (MED-EL, Innsbruck, Austria). The CI system comprises an external Audio Processor (both cases received the Sonnet, MED-EL, Innsbruck, Austria), and the implanted component contains a magnet, which holds the external component in place above the implant. The Standard Electrode features 24 platinum electrode contacts with a stimulation range of 26.4 mm.

### 2.2. Pre-Op Examinations

- Nasopharyngeal swab analysis for SARS-CoV-2;
- Local otoscopy and otomicroscopy exams;
- Pure-tone audiometry (PTA);
- Tympanogram;
- Functional vestibular testing was performed via video head impulse test (vHIT);
- Auditory brain stem response (ABR).

### 2.3. Intraop Examinations

- Perilymph was taken from the cochlea and sent for virological analysis.

### 2.4. Postop Examinations

- Pure-tone audiometry (PTA).

## 3. Results

### 3.1. CASE 1

We present a 45-year-old female patient who was referred to our ENT clinic for a further evaluation of her symptoms of sudden hearing loss in both ears, tinnitus, instability, and dizziness, which were most pronounced in low-light environments. The patient had no previous history of noise exposure, head trauma, exposure to ototoxic drugs, autoimmune, neurological, or audio-vestibular diseases.

A routine nasopharyngeal swab analysis for SARS-CoV-2 was negative, but IgG antibodies were tested positive, indicating a previous infection. During hospital admission, a clinical ENT examination and local otoscopy and otomicroscopy exams were performed, showing no pathological processes in the external ear and tympanic membrane. The laboratory findings were in the normal range.

**Figure 1:**
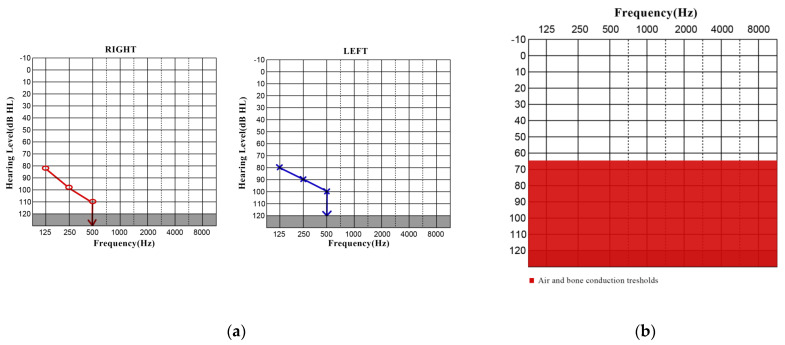
(**a**) Pure-tone audiogram indicating the pre-op bilateral profound SNHL. (**b**) Cochlear implant indication criteria (MED-EL, Innsbruck, Austria).

The pure-tone audiometry (PTA) showed profound SNHL in both ears, and the respective cochlear implant indication range is shown in Figure 1a,b, respectively.

The tympanogram had a normal bell-shaped curve, with a top between +50 and −99 daPa. An acoustic reflex was absent in both ears. Functional vestibular testing was performed. The video head impulse test (vHIT) for the horizontal semicircular canal was positive on both sides (covert saccades and low VOR gain bilaterally: left 0.48 and 0.36 right), without spontaneous or gaze-evoked nystagmus.

The auditory brain stem response (ABR) did not show a clear answer in the highest levels of stimulus (100 dB). The bithermal infrared caloric nystagmography test described total bilateral areflexia. No cervical vestibular evoked myogenic (cVEMP) potentials were obtained at 90 or 100 dB of stimulation (no wave occurrence).

**Figure 2:**
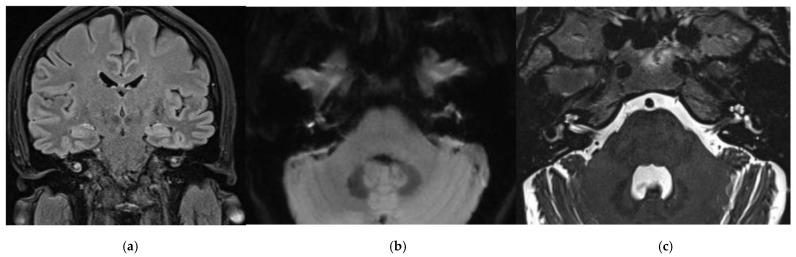

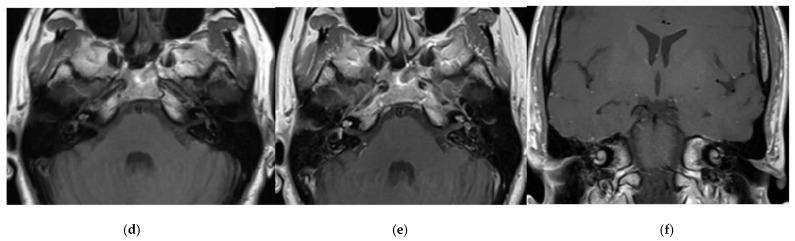
Magnetic resonance images. Hyperintense signal of cochlea, vestibule, and part of semicircular canals on both sides, more prominent on the right side, indicating labyrinthitis and signs of possible bleeding in the membranous labyrinth in axial T2W SPACE (**a**), coronal FLAIR (**b**), axial DWI (**c**), axial precontrast T1WI (**d**), and axial (**e**) and coronal postcontrast T1WI (**f**). MRI findings.

A diagnosis of bilateral audio-vestibular disorder was performed. A CT scan showed a discrete change in the white matter in the front left at the border of the corona radiata and the centrum ovale near the cortico-subcortical border. The MRI findings indicate labyrinthitis more to the right side with signs of possible hemorrhage in the membranous labyrinth (Figure 2a–f).

Urgent therapy with a high dose of oral corticosteroids was included (Prednisone, 7 days with a dose of 60 mg and 7 days with a reduced dose, with additional conservative therapy). A controlled audio-vestibular assessment was performed 14 days after starting therapy. There was no improvement in the aided hearing threshold measured in overhead headphones on PTA and no improvement in vestibular function on the vHIT and bithermal caloric test.

**Figure 3:**
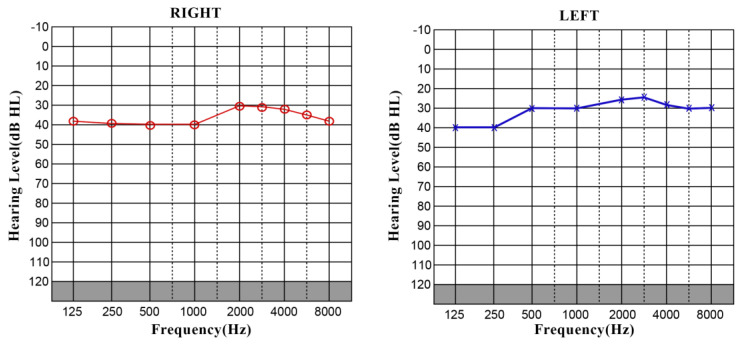
Postoperative aided PTA measured in overhead headphones revealed mild SNHL on both ears (125–4000 Hz 45 dB).

Due to a poor therapeutic response, cochlear implant surgery was performed on both ears, without any postoperative complications. The intraoperative findings had shown initial signs of fibrotic changes in the tympanic scale. The perilymph was taken from the cochlea and sent for a virological analysis that was negative for the presence of SARS-CoV-2 virus. The intraoperative telemetry showed a good range in 8 of the 12 channels in the right ear, and in 10 of 12 channels in the left ear. The postoperative PTAs showed improvements in the speech frequencies (Figure 3).

### 3.2. CASE 2

The second case was a 46-year-old male patient with initial symptoms of dizziness and sudden hearing loss in both ears, which occurred 3 weeks after a COVID-19 infection was verified. He had no previous neurological nor ear symptoms. He has been undergoing diabetes therapy for several months. Immediately after he finished his COVID-19 infection treatment, he was admitted in our clinic for further evaluation. The otomycroscopy exam showed no pathological changes. The facial nerve function remained normal. The laboratory findings were in the reference range.

**Figure 4:**
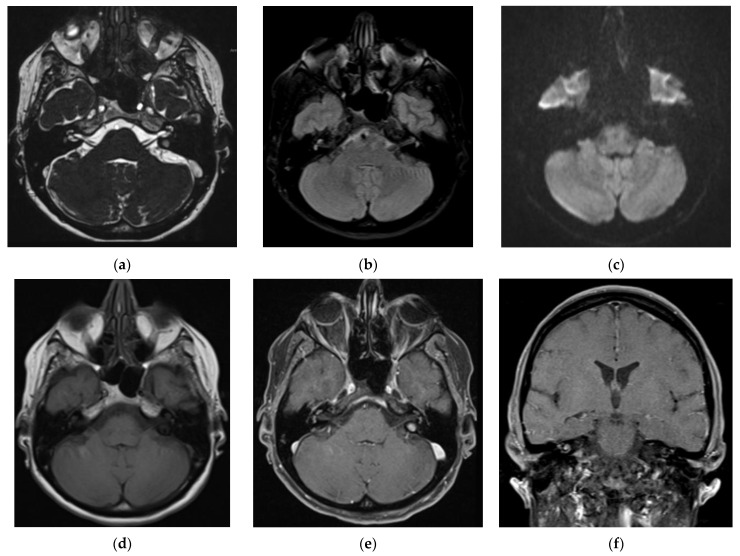
Magnetic resonance images. Hyperintense signal of cochlea, vestibule, and semicircular canals on both sides, more prominent on the right side, in axial T2W SPACE (**a**), coronal FLAIR (**b**), and axial (**e**) and coronal postcontrast T1WI (**f**), without alteration of signal intensity on axial DWI (**c**) and axial precontrast T1WI (**d**).

The MRI revealed a pair of chronic micropangiopathic changes in the convexity frontally supracortical, with reductive changes in the convexity of the parenchyma bifrontally and a graceful cavum septi pelucidi/cavum verge. An arachnoid cyst in the frontal interhemispheric parasagittal left with falx was found, and a second mediotemporal basal was found without significant compressive manifestations (Figure 4).

**Figure 5:**
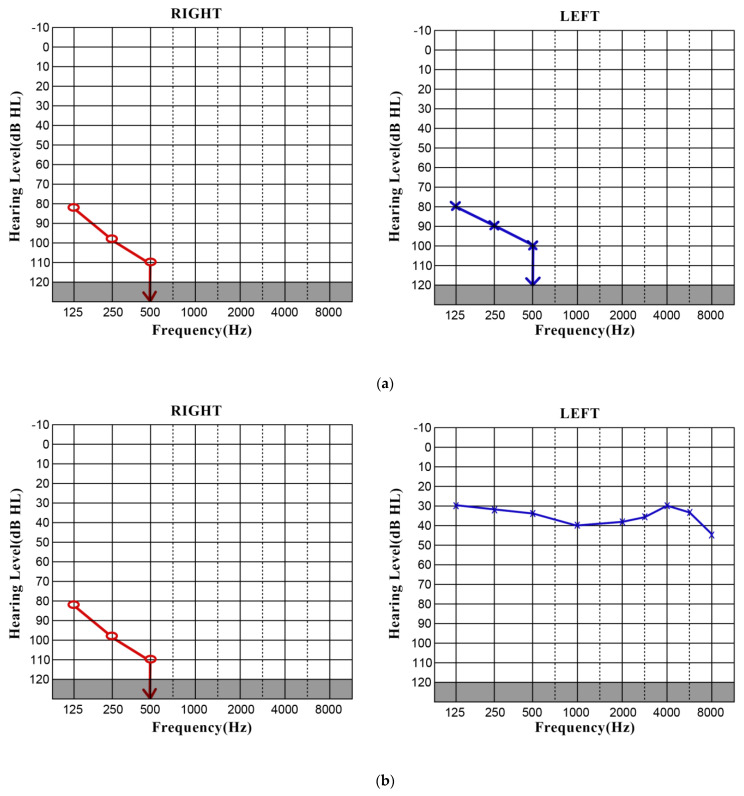
(**a**) Pure-tone audiogram (PTA) indicating the pre-op bilateral profound SNHL. (**b**) Postoperative aided PTA measurement on the left side. Unaided PTA measurements were performed on the right side as the right ear was not treated, and PTA remained the same as before the left ear surgery.

The temporal bone on the CT scan was seen without inflammatory or expansive processes. Pure-tone audiometry was performed, and bilateral profound SNHL was diagnosed (Figure 5a). There was no presence of spontaneous or gaze-evoked (fixation) nystagmus. The HIT was positive bilaterally. On vHIT, an unmeasurable gain in all six semicircular canals was obtained. The bithermal caloric test showed bilateral areflexia. There was no response on the cVEMP. After the intra-tympanic injection of dexametasone (4 mg/mL) with no significant clinical improvement, and an exhaustive preoperative diagnosis, a cochlear implantation in the left ear was performed on the fourth hospital day. The initial intraoperative telemetry showed that all 12 channels were in use with good ranges. The patient refused to undergo a cochlear implant on the right ear, and the right ear was not operated on, despite having an improved PTA threshold postoperatively (Figure 5b).

Audiological rehabilitation was performed in both patients, and good results were obtained. Both patients reported satisfaction with their new sound perception.

## 4. Discussion

Viral SNHL, tinnitus, vertigo, dizziness, or imbalance are presumed to be based on a direct invasion and damage to inner ear structures, including the organ of Corti (OC) and the vestibulocochlear nerve [7], and/or through immune-mediated damage and inflammation, including neuroinflammation [8,9], and/or the reactivation of a latent virus within the inner ear [10]. The incidence of SNHL is 5–20 per 100,000 [11]. Nearly all cases are unilateral, and less than 2% have bilateral involvement [12]. Compared with unilateral SNHL, bilateral hearing loss has a relatively worse prognosis [13]. The accompanying symptoms are tinnitus and dizziness. The potential discoverable etiologies are infections, autoimmune diseases, traumatic events, vascular lesions, neoplastic processes, metabolic problems, and neural causes. In one meta-analysis of 23 studies of SNHL, the most frequent causes identified were infectious (13%) [14]. In most cases, hearing loss results from damage to hair cells or other cochlear parts. Sometimes, the damage is irreversible. Many viruses have been implicated in SNHL onset like herpes simplex virus, varicella zoster, enteroviruses, mumps, and influenza [14]. One of the main theories of the pathophysiology of viral SNHL is that viral infection or viral reactivation in the inner ear causes cochlear inflammation and damage to the relevant structures of the inner ear. Significant levels of serum antiviral antibodies have been isolated from the serum of patients with SNHL [15]. The temporal bones from patients with idiopathic sudden SNHL show histological patterns similar to those seen in viral labyrinthitis, including the atrophy of the organ of Corti, tectorial membrane, stria vascularis, and vestibular end organ [16]. Additionally, viruses are known to infect the middle ear and typically cause conductive hearing loss due to middle-ear effusion. While coronaviruses are common causes of middle ear infection [17], their roles in inner ear infection have not been systematically investigated.

Due to the corona virus pandemic, various manifestations of the disease have been reported in the past two years, including SNHL. The exact incidence is not known because previous research was mainly limited to case reports. A systematic review and meta-analysis performed by Tang et al. (2023) using a random-effects model showed that the overall prevalence of hearing loss among COVID-19-positive patients from different countries was 8.2% (95% CI, 5.0–12.1) [18]. The study by Mehraeen et al. reported on an incidence rate of 0.2–7.6% [19]. The literature related to COVID-19 infection is growing, and several different etiology factors for SNHL have been described: from the direct damage of the cochleovestibular system to neurotropic invasion or inflammatory induced reaction. According to a recent systematic review and meta-analysis of cohort studies during the 2020–2021 period, cross-sectional observational studies revealed SNHL in audiological testing in more than half of the population [20]. Edwards et al. reported bilateral profound SNHL shortly after symptomatic infections with COVID-19 [21]. Rhman SB et al. presented a patient who had unilateral moderate to severe SNHL as the only symptom of COVID-19 infection with clinical improvement after the intratympanic injection of 40 mg/kg methylprednisolone [22]. Asfour et al. described a case of a patient with unilateral deep SNHL after a COVID-19 infection who underwent cochlear implantation with a good postoperative response [23]. In the case report of Gerstacker et al., in a patient with acute bilateral profound SNHL treated for severe pneumonia caused by COVID-19 infection, cochlear implantation was performed after systemic and intratympanic steroid therapy and did not show clinical improvement, similar to our two presented cases [24]. A limitation of our study is that lateralization postop was not investigated; despite the fact that one of the two cases was bilaterally implanted, and the other one only had a unilateral implant, and the contralateral side stayed untreated, further studies are required to test the lateralization benefit and difference in those two subjects, especially since it is known that neural processes underlying both perception and covert production speech and singing activate overlapping brain regions [25]. It is well known that in post-lingually deaf adult CI users, a high progress of speech recovery is observed during the first year after CI, but there is a large range of variability in the CI outcomes and a temporal evolution of recovery [26].

There is heterogeneity in COVID-19 severity and the timing of audio-vestibular symptoms, with the timing of audio-vestibular symptoms ranging from being the first indication of COVID-19 within 3–12 days and between 4 and 12 weeks after reporting COVID-19 symptoms [20]; hence, making decisions towards implantations is difficult, but due to the possible irreversible ossification and fibrosis of the cochlea seen in many subjects, fast decisions towards implantation to allow for better postoperative results are necessary.

Fibrosis and possible ossification may be because of ACE2, which is also expressed on the epithelial cells of the respiratory mucosa, which continues through the Eustachian tube into the middle ear, and another potential mechanism is the direct invasion of the cochlear nerve and/or labyrinth [27,28]. The hyperintensive signal of cochlea in our patient’s MRI could be ascribed to virus-related immune-mediated inflammation, which is also presented in some other studies [29]. The study by Fancello et al. reported MRI findings such as inner ear damage, bilateral intra-labyrinthine hemorrhage, bilateral cochlear inflammation, and cochlear fibrosis, which is similar to what we discovered in our patients [29]. Another relevant reason for those changes in the cochlea can refer to intralabyrinthine hemorrhage, which has been documented in a case of bilateral SNHL, and vertigo, which was documented in a patient with a history of SARS-CoV-2 infection [8].

In terms of the diagnostic principles, precise anamnesis including the detection of ethiological factors like previous audio-vestibulological events, noise exposure, head trauma, autoimmune disease, ototoxic drugs, Meniere disease, and ear malformations need to be taken into account. Another etiologiy for acute deafness might be the thromboembolic potential of the virus. Every patient should have an MRI to rule out a retrocochlear lesion, which is important when selecting patients for cochlear implantation.

Unfortunately, there are no defined protocols for the treatment of SNHL so far. The results are often inconclusive and refer to the case series and small trials. Corticosteroids are thought to improve idiopathic sudden SNHL by reducing inflammation and edema in the inner ear [8]. Intratympanic corticosteroids are used frequently in management, whichleads to higher perilymph levels of steroids than systemic therapy, but they do not achieve increased levels of the drug in the circulation and are used mainly in patients with contraindications or in patients who did not respond well to the initial systemic steroid treatment. For idiopathic sudden SNHL, 45 to 65% of patients will regain their pre-loss hearing thresholds even without therapy [30]. Rhman et al. presented a COVID-19-positive patient with severe unilateral sudden SNHL who had a hearing improvement after an intra-tympanic injection of methylprednisolone [22]. Our second patients had a similar treatment approach but showed no changes in the hearing threshold levels. Cochlear implantation is a form of hearing rehabilitation for bilateral moderate to profound SNHL when there is no help from hearing aids. Cochlear implantation allows for the direct electrical stimulation of the auditory nerve, and hence, the preservation of central auditory nerve structures is of particular importance when selecting candidates for cochlear implantation. Due to the possible irreversible ossification and fibrosis of the cochlea, it is necessary to perform implantation as soon as possible for better postoperative results.

Further research is needed and will hopefully shine light on many currently unknown or poorly understood impacts of COVID-19. Unfortunately, this area of research is limited to emerging studies with small population sizes and case reports, and at present, little is known about the risk factors for SNHL following COVID-19 infection, so it is difficult to alter hearing screening protocols accordingly and implement early treatment with corticosteroids. It is vital that SNHL will be rigorously investigated among people who have active or recently recovered COVID-19 infections, as the risk of permanent audio-vestibular symptoms cannot be disregarded in the future.

## 5. Conclusions

Only a few studies, mainly case reports, relating to the incidence of SNHL and COVID-19 infection are available, but there is an increase in interest in this field. Several main questions like the mechanisms of injury and treatment protocols remained undefined. In SNHL, with above 70 dB of hearing loss, without a response to steroid therapy and no benefits of hearing aids, CI is indicated. Both patients presented here reported great satisfaction with their new sound perception following cochlear implantation. Proper patient selection is necessary with the exclusion of retro cochlear damage to the auditory system. Further investigation with a larger number of participants will lead to more precise conclusions.

## Data Availability

Data are contained within the article.
